# DNMT3A-mediated silence in ADAMTS9 expression is restored by RNF180 to inhibit viability and motility in gastric cancer cells

**DOI:** 10.1038/s41419-021-03628-5

**Published:** 2021-04-30

**Authors:** Weilin Sun, Gang Ma, Li Zhang, Pengliang Wang, Nannan Zhang, Zizhen Wu, Yinping Dong, Fenglin Cai, Liqiao Chen, Huifang Liu, Han liang, Jingyu Deng

**Affiliations:** 1 Department of Gastroenterology, Tianjin Medical University Cancer Institute and Hospital, National Clinical Research Center for Cancer; Key Laboratory of Cancer Prevention and Therapy, Tianjin; Tianjin’s Clinical Research Center for Cancer, Tianjin, 300060 China; 2 Affiliated Cancer Hospital & institution of Guangzhou Medical University, Guangzhou, China

**Keywords:** Gastric cancer, Cell invasion

## Abstract

ADAMTS9 belongs to the ADAMTS (a disintegrin and metalloproteinase with thrombospondin motifs) protein family, and its expression is frequently silenced due to promoter hypermethylation in various human cancers. However, the underlying mechanisms remain largely unknown. In this study, we investigated the inhibitory effects of ADAMTS9 on gastric cancer (GC) cells. We initially examined ADAMTS9 protein level in 135 GC and adjacent normal tissue pairs, showing that ADAMTS9 was strikingly decreased in the malignant specimens and patients with low ADAMTS9 expression exhibited more malignant phenotypes and poorer outcome. ADAMTS9 expression was restored in AGS and BGC-823 cells, which then markedly suppressed cellular viability and motility in vitro and in vivo. As ADAMTS9 was enriched in the nuclei of gastric mucosal cells, RNA-sequencing experiment showed that ADAMTS9 significantly altered gene expression profile in BGC-823 cells. Additionally, DNA methyltransferase 3α (DNMT3A) was identified to be responsible for the hypermethylation of ADAMTS9 promoter, and this methyltransferase was ubiquitinated by ring finger protein 180 (RNF180) and then subject to proteasome-mediated degradation. In conclusion, we uncovered RNF180/DNMT3A/ADAMTS9 axis in GC cells and showed how the signaling pathway affected GC cells.

## Introduction

Aberrant promoter DNA hypermethylation is a well-defined epigenetic hallmark in all human tumor types. Dense promoter methylation controls gene expression temporally and spatially, which further facilitates initiation and progression of cancer^1,2^. During neoplasia, the expressions of critical tumor suppressor genes, such as VHL, CDKN2A, and BRCA1/2, are silenced due to increased DNA methylation in promoter regions, conferring selective advantages to preneoplastic cells^1,3^. In recent years, next-generation sequencing assays demonstrated that DNA methylation condition markedly changed on a whole genome scale in cancer cells, thereby holding promise for identifying powerful diagnostic, prognostic, and therapeutic biomarkers^4^. However, only few DNA methylation-related biomarkers have been translated into commercially assays until now, such as SEPT9 for colorectal cancer, GSTP1 for prostate cancer^5^. Gastric cancer (GC) is the fifth most common malignant tumor and third leading cause of cancer-related death^6^. A growing body of data strongly suggests that altered DNA methylome is essential for GC tumourigenesis and progression, and several genes have been identified to be associated with chemoresistance, lymph node (LN) metastasis, and poor survival^7^. However, the functions and mechanisms of a plethora of hypermethylated genes in GC remain largely unknown.

A disintegrin-like and metalloproteinase with thrombospondin motifs (ADAMTS) superfamily contains 26 members, which belongs to ADAMTS protease family and ADAMTS-like protein family, respectively^8^. ADAMTS protease family consists of 19 secreted zinc metalloproteases, whose substrates are primarily extracellular matrix (ECM) components^8^. Accumulating evidence showed that ADAMTS proteins were essential to sustain embryonic development and tissue homeostasis^8^. Pro-tumor and antitumor effects of ADAMTS proteins have been uncovered in distinct cancer settings. ADAMTS1 acts as a pro-tumor delegate in ADAMTS protease family, which was overexpressed in pancreatic/breast cancer and facilitated metastasis^9–11^. On the other hand, the expressions of several ADAMTS genes, such as ADAMTS5/8/18, were epigenetically silenced as their promoters were densely methylated^12^. ADAMTS9 is an additional tumor suppressing protein, which was markedly decreased in esophageal, nasopharyngeal, gastric, colorectal, pancreatic cancer as well as multiple myeloma^13–18^. Restored ADAMTS9 expression abrogated viability in GC cells via reducing activity of AKT/mTOR pathway^15^. These observations highlighted the antitumor functions of ADAMTS9; however, the precise molecular mechanisms underlying the roles of ADAMTS9 in GC have not been completely understood.

In this study, we investigated the inhibitory functions of ADAMTS9 in GC, showing that ADAMTS9 expression was decreased in malignant tissues and this low level was positively associated with advanced stage and poor overall survival (OS) in patients with GC. Similarly, we found that ADAMTS9 attenuated malignant phenotypes in GC cells. As ADAMTS9 was partially localized in the nuclei, we performed RNA-sequencing assay and showed that this protein altered the expressions of dozens of genes in BGC-823 cells, some of which are closely related with viability and motility in GC cells. We identified that DNMT3A dictated the promoter hypermethylation of ADAMTS9, and the activity of this methyltransferase remained intact largely because RNF180, as its E3 ligase, was absent in GC cells. Together, we provided a new mechanistic insight into the function of ADAMTS9 in GC.

## Materials and methods

### Patients and tissue samples

The tissue microarrays (TMAs, Cat No. T14-501 TMA1-3) used in this study were composed of 135 pairs of GC and adjacent non-tumor tissues, which were derived from patients with GC receiving curative gastrectomy in Department of Gastroenterology, Tianjin Medical University Cancer Institute and Hospital (Tianjin, China) between August 2004 and December 2007. These TMAs were engineered by Shanghai Outdo Biotech Company (Shanghai, China). Follow-up was performed every 3–6 months and completed in September 2012. The median was 34.0 months (Range: 2–75 months). Clinical data including B-mode ultrasound, CT scans, chest X-ray, and endoscopy available were collected.

Additionally, 16 pairs of randomly selected GC and adjacent non-tumor tissues between July and December 2018 were used for RNA extraction. All these enrolled patients did not receive neoadjuvant therapy before gastrectomy and their clinicopathological characteristics are summarized in Supplementary Table S1 and S2, respectively. Patient consents were obtained and all experiments related to these specimens and clinical data were approved by Institutional Research Ethics Committee of Tianjin Medical University Cancer Institute and Hospital (Tianjin, China).

### RNA-sequencing and analysis

BGC-823 cells were stably transfected with pCNDA3.1-ADAMTS9 or empty pCDNA3.1 vector. Total RNA was then extracted using TRIzol reagent (Invitrogen). RNA integrity was examined using an Agilent 2100 Bioanalyzer (Agilent Technologies, Santa Clara, CA, USA). Libraries were constructed using TruSeq Stranded mRNA LT Sample Prep Kit (Illumina, San Diego, CA, USA) according to the manufacturer’s instructions. Hierarchical cluster analysis of differentially expressed genes (DEGs) was conducted to explore gene expression patterns. Gene ontology (GO) enrichment and Kyoto Encyclopedia of Genes and Genomes (KEGG) pathway enrichment analysis of DEGs were performed using R software. Transcriptome sequencing and analysis were completed by OE Biotech Co., Ltd. (Shanghai, China).

### MassARRY analysis of the methylation level of ADAMTS9

Genomic DNA of BGC-823 and AGS cells were extracted using QIAamp DNA Mini Kit (Qiagen). Then, the methylation of ADAMTS9 promoter was detected using an MassARRAY analyzer (Agena Bioscience, San Diego, CA, USA). EpiTYPER software was used to calculate the methylation ratio.

Other experimental and statistical methods are available in the Supplementary dataset.

## Results

### Loss of ADAMTS9 expression in GC indicates increased LN metastasis and poor prognosis

**Fig. 1:**
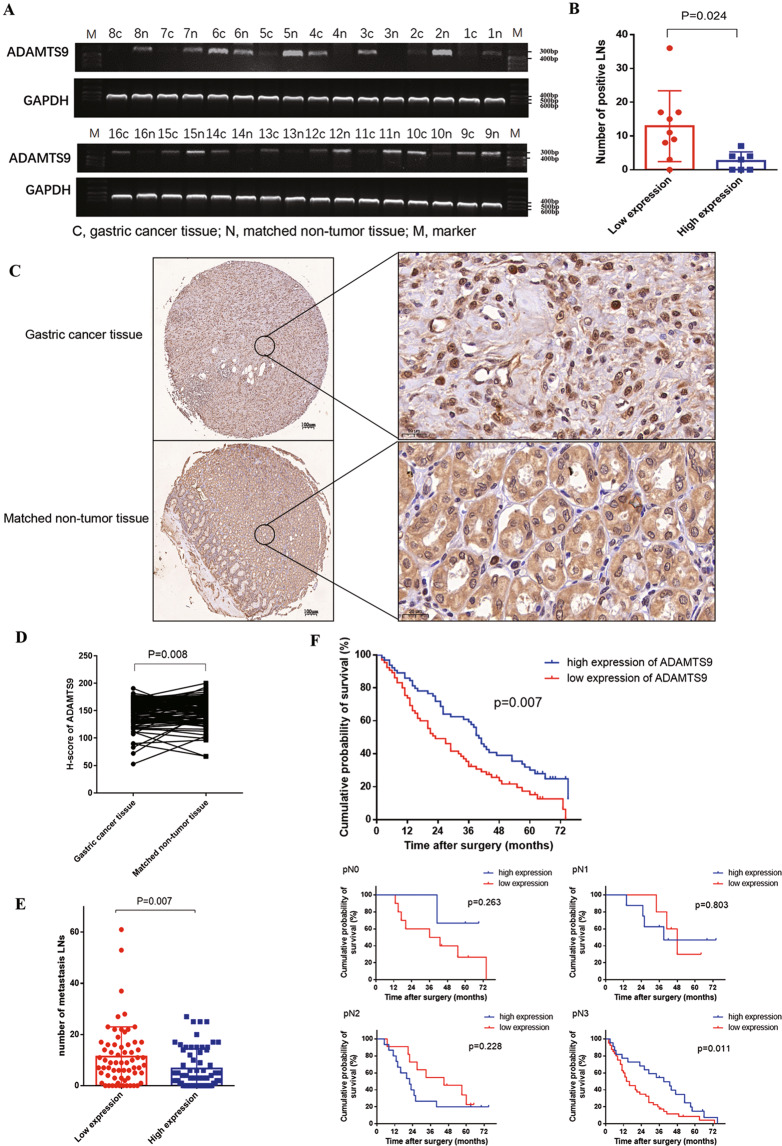
Low expression of ADAMTS9 in GC tissues was associated with the more metastatic LNs and the poorer survival outcomes of GC patients. **A** mRNA expression levels of ADAMTS9 in GC tissues and matched non-tumor tissues were analyzed by reverse transcriptase PCR assay. **B** Correlation analysis was performed between ADAMTS9 mRNA expression and metastatic LNs. **C** Protein expression levels of ADAMTS9 in GC tissues and matched adjacent non-tumor tissues were analyzed by IHC assay. **D** ADAMTS9 protein was significantly decreased in GC tissues. **E** Low expression level of ADAMTS9 was closely associated with more metastatic LNs in GC patients. **F** Keplan–Meier survival analysis was performed in 129 GC patients and stratification by pN stage.

We examined ADAMTS9 mRNA levels in 16 pairs of GC and adjacent normal tissues, showing that ADAMTS9 mRNA levels were markedly decreased in nine tumor tissues compared with non-tumor counterparts (Fig. 1A). Analyzing the potential correlation between clinicopathological characteristics and mRNA levels of ADAMTS9, we found that ADAMTS9 expression was negatively associated with pN stage (*P* = 0.044) and Lauren type (*P* = 0.019) (Supplementary Table S1). In addition, patients with low ADAMTS9 mRNA expression were at high risk for increased LN metastasis (12.89 ± 3.49 vs 2.571 ± 1.020, *P* = 0.024, Fig. 1B).

We also detected ADAMTS9 protein using immunohistochemistry (IHC), showing that ADAMTS9 was enriched in the cytoplasm and nucleus of gastric mucosal cells (Fig. 1C). As shown in Fig. 1D, *H*-scores demonstrated that ADAMTS9 protein levels were significantly lower in GC tissues relative to in matched normal ones (*N* = 111, *P* = 0.008, Fig. 1D). These patients with GC were then categorized into low/high group based on median *H*-score of ADAMTS9, and the clinicopathological characteristics of the two groups are summarized in Supplementary Table 2. Univariate analysis showed that patients with low ADAMTS9 expression exhibited advanced pN stage (*P* = 0.018) and decreased LN metastasis compared with those in the ADAMTS9-high group (11.38 ± 11.64 vs 6.73 ± 7.63, *P* = 0.007; Fig. 1E).

To determine the prognostic value of ADAMTS9 expression in patients with GC, we performed Kaplan–Meier analysis. As shown in Fig. 1F, GC patients with low ADAMTS9 expression had a poorer 5-year survival rate compared to those with high expression (*P* = 0.007). Multivariate cox regression analysis was further performed and ADAMTS9 was identified to be an independent predictor of prognosis (HR 1.602, 95% CI 1.069–2.399, *P* = 0.022) (Table 1). We also evaluated the importance of node status with respect to survival outcomes through pN stage stratification, finding that low ADAMTS9 expression indicated poor survival outcomes in patients with pN3 stage (*P* = 0.011, Fig. 1F). Together, these results suggested that loss of ADAMTS9 probably promoted GC progression.

**Table 1:** Univariate and multivariate Cox proportional hazard models for overall survival of gastric cancer patients.

Predictor	Univariate analysis		Multivariate analysis		AIC	BIC
	HR(95% CI)	*P*	HR (95% CI)	*P*		
**Gender**						
Female vs male	1.372 (0.905–2.079)	0.136				
**Age**						
≥60 vs <60	1.514 (1.012–2.245)	0.039^*^	1.528 (1.005–2.323)	0.047^*^	74.730	91.889
**Tumor size**						
≥5 cm vs <5 cm	1.888 (1.250–2.851)	0.003^**^	1.591 (1.041–2.430)	0.032^*^	72.686	89.844
**Tmuor location**						
Middle 1/3 vs up 1/3	1.232 (0.588–2.584)	0.580				
Low 1.3 vs up 1/3	0.948 (0.545–1.649)	0.851				
>2/3 stomach vs up 1/3	1.235 (0.661–2.307)	0.508				
**Lauren type**						
Diffuse vs intestinal	1.144 (0.677–1.934)	0.615				
Mixed vs intestinal	1.621 (0.637–4.123)	0.311				
**Borrmann type**						
II vs I	0.552 (0.073–4.173)	0.565				
III vs I	0.385 (0.052–2.847)	0.350				
IV vs I	0.512 (0.064–4.113)	0.529				
**pT stage**						
pT3 vs pT2	0.547 (0.164–1.821)	0.326				
pT4 vs pT2	0.788 (0.381–1.628)	0.519				
**pN stage**						
pN1c vs pN0	0.958 (0.397–2.315)	0.925	0.784 (0.319–1.929)	0.597	81.240	92.680
pN2 vs pN0	1.745 (0.911–3.341)	0.093	1.480 (0.758–2.889)	0.251		
pN3 vs pN0	2.828 (1.638–4.883)	<0.001^***^	2.231 (1.273–3.912)	0.005^**^		
**Expression of ADAMTS9**						
Low vs high	1.732 (1.166–2.573)	0.009^**^	1.602 (1.069–2.399)	0.022^*^	71.555	88.714

*AIC* akaike information criterion, *BIC* Bayesian information criterion.

^*^P < 0.05; ^**^P < 0.01; ^***^P < 0.001.

### ADAMTS9 impairs GC cell viability in vitro and in vivo

**Fig. 2:**
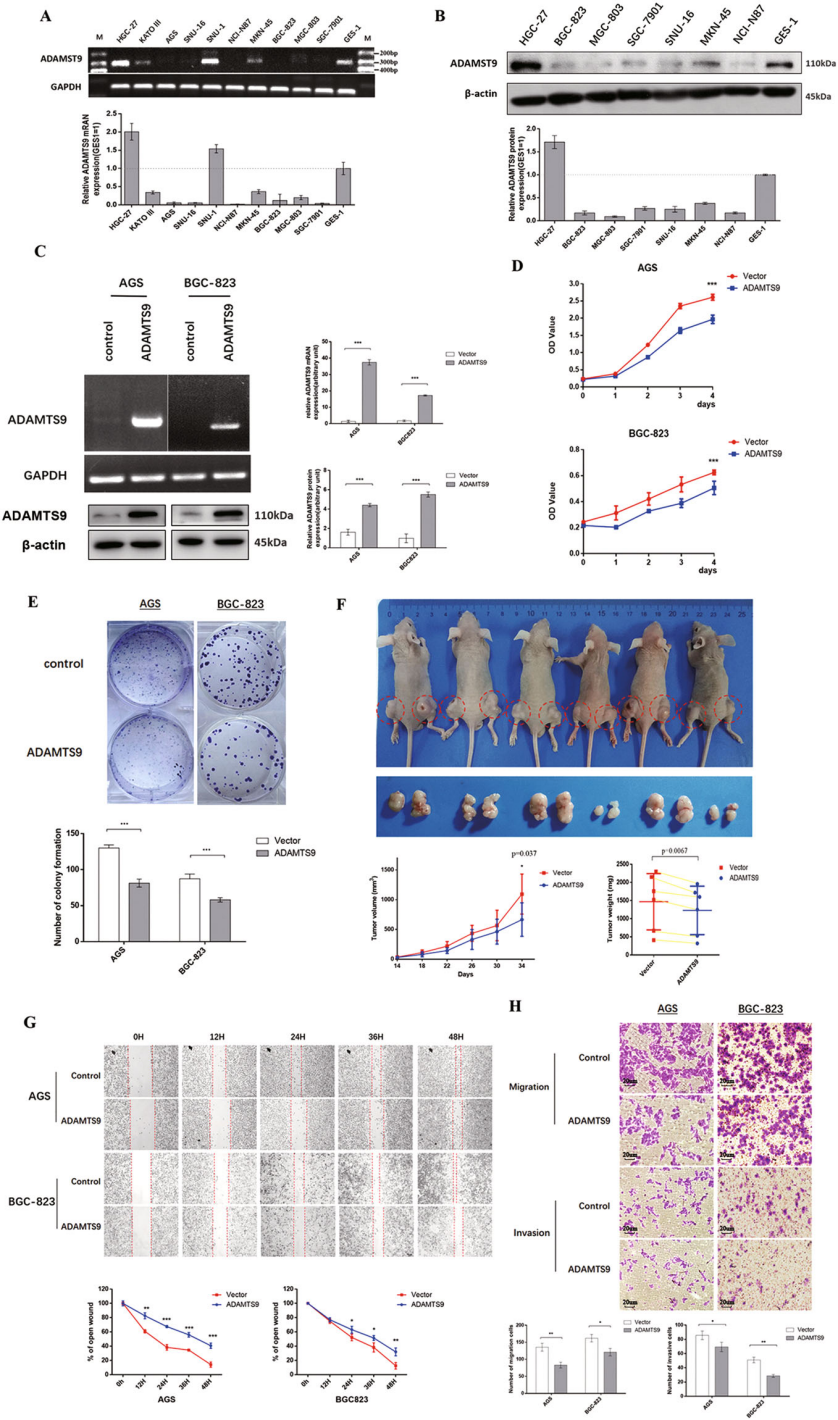
ADAMTS9 attenuated malignant phenotypes in AGS and BGC-823 cells. **A** ADAMTS9 expression levels were examined by reverse transcriptase PCR assay; **B** and western blotting assay. **C** Expression levels of ADAMTS9 in AGS and BGC-823 cells transfected with ADAMTS9 overexpression plasmid and empty plasmid were analyzed by reverse transcriptase PCR and western blotting assay. **D** ADAMTS9 inhibits the cell growth in AGS and BGC-823 cells. **E** ADAMTS9 suppresses the colony formation in AGS and BGC-823 cells. **F** ADAMTS9 inhibited growth of tumors in vivo. Tumor growth curves and tumor weight shows the suppressive effect of ADAMTS9 in vivo. **G** ADAMTS9 impairs motile capacity in AGS and BGC-823 cells detected by wound healing assay (magnification 40×); **H** and by transwell assay (magnification 100×).

To investigate the function of ADAMTS9 in GC cells, we firstly evaluated its expression in a panel of human GC cell lines and an immortalized human gastric epithelial cell line GES-1, finding that mRNA and protein levels of ADAMTS9 were markedly decreased in most GC cell lines compared with in GES-1 cells (Fig. 2A, B). Then, we restored ADAMTS9 expression in AGS, BGC-823, and SGC-7901 cells, the increased expression which was confirmed via RT-PCR and immunoblot (Fig. 2C and Supplementary Fig. S1A). We found that ADAMTS9 could inhibit viability in AGS, BGC-823, and SGC-7901 cells as shown in CCK8 assays (Fig. 2D and Supplementary Fig. S1B). Likewise, this protein could suppress the capacity of colony formation in these three GC cell lines (Fig. 2E and Supplementary Fig. S1C). We also subcutaneously inoculated BGC-823 cells with ADAMTS9 overexpression and the control ones into six male Balb/c nude mice and detected growth of the tumor masses within 34 days. As shown in Fig. 2F, ADAMTS9-overexpressing BGC-823 cells exhibited markedly decreased growth rate relative to the control cells. Meanwhile, the weights of the harvested tumor masses demonstrated that ADAMTS9 inhibited the proliferation of BGC-823 cells in vivo (Fig. 2F).

### ADAMTS9 undermines GC cell motility in vitro

As described before, ADAMTS9 deletion was positively associated with LN metastasis. Thus, we investigated the effect of ADAMTS9 on motility of AGS, BGC-823, and SGC-7901 cells. Wound healing assays showed that ADAMTS9 significantly reduced the migration distances compared to that of control cells (AGS cells at 48 h, *P* < 0.001; BGC-823 cells at 48 h, *P* = 0.009; and SGC-7901 cells at 36 h, *P* < 0.001; Fig. 2G and Supplementary Fig. S1D). Moreover, transwell assays showed that ADAMTS9 impaired migration and invasion in GC cells (migration assay: AGS, *P* = 0.003; BGC-823, *P* = 0.010; and SGC-7901, *P* = 0.010; invasion assay: AGS, *P* = 0.035; BGC-823, *P* = 0.010; and SGC-7901, *P* = 0.004; Fig. 2H and Supplementary Fig. S1E). Together, these results demonstrated that ADAMTS9 attenuated malignancy in GC.

### ADAMTS9 alters the gene expression profile in GC cells

**Fig. 3:**
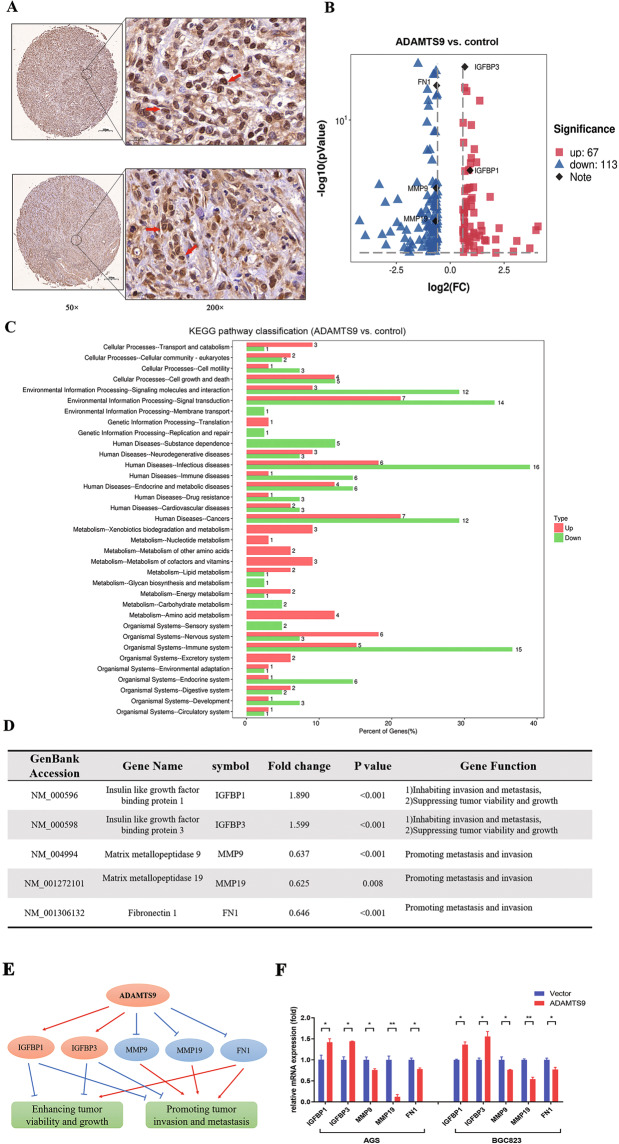
Downstream molecular of ADAMTS9 in GC were identified by using the RNA-sequencing method. **A** ADAMTS9 is enriched in the nuclei of gastric mucosal cells. **B**, **C** The volcano plots and KEGG enrichment plots were generated from mRNA sequencing analysis of ADAMTS9-overexpressing BGC-823 cells and control cells transfected with empty plasmid. **D**, **E** Downstream potent molecular events were identified for impairing viability, proliferation, and motile capacity. **F** The downstream DEGs were validated by the qPCR in AGS and BGC-823 cells.

As IHC results showed that ADAMTS9 was positively stained in the nuclei of gastric mucosal cells (Fig. 3A), we speculated that this protein might be implicated in regulating gene expression. Thus, we performed next-generation sequencing to analyze mRNA profile in the control and the ADAMTS9-overexpressing BGC-823 cells, showing that the expressions of 183 genes (DEGs) in total were significantly changed (FC > 1.5) (Fig. 3B). The KEGG pathway enrichment of these DEGs was shown in Fig. 3C. Among these DEGs, five representatives (IGFBP1/3, MMP9/19, and FN1) and their functions were summarized in 3D and 3E. All these five genes expressions were further confirmed using qPCR in AGS and BGC-823 cells (Fig. 3F).

### DNMT3A is primarily responsible for increasing methylation in promoter of ADAMTS9 gene

**Fig. 4:**
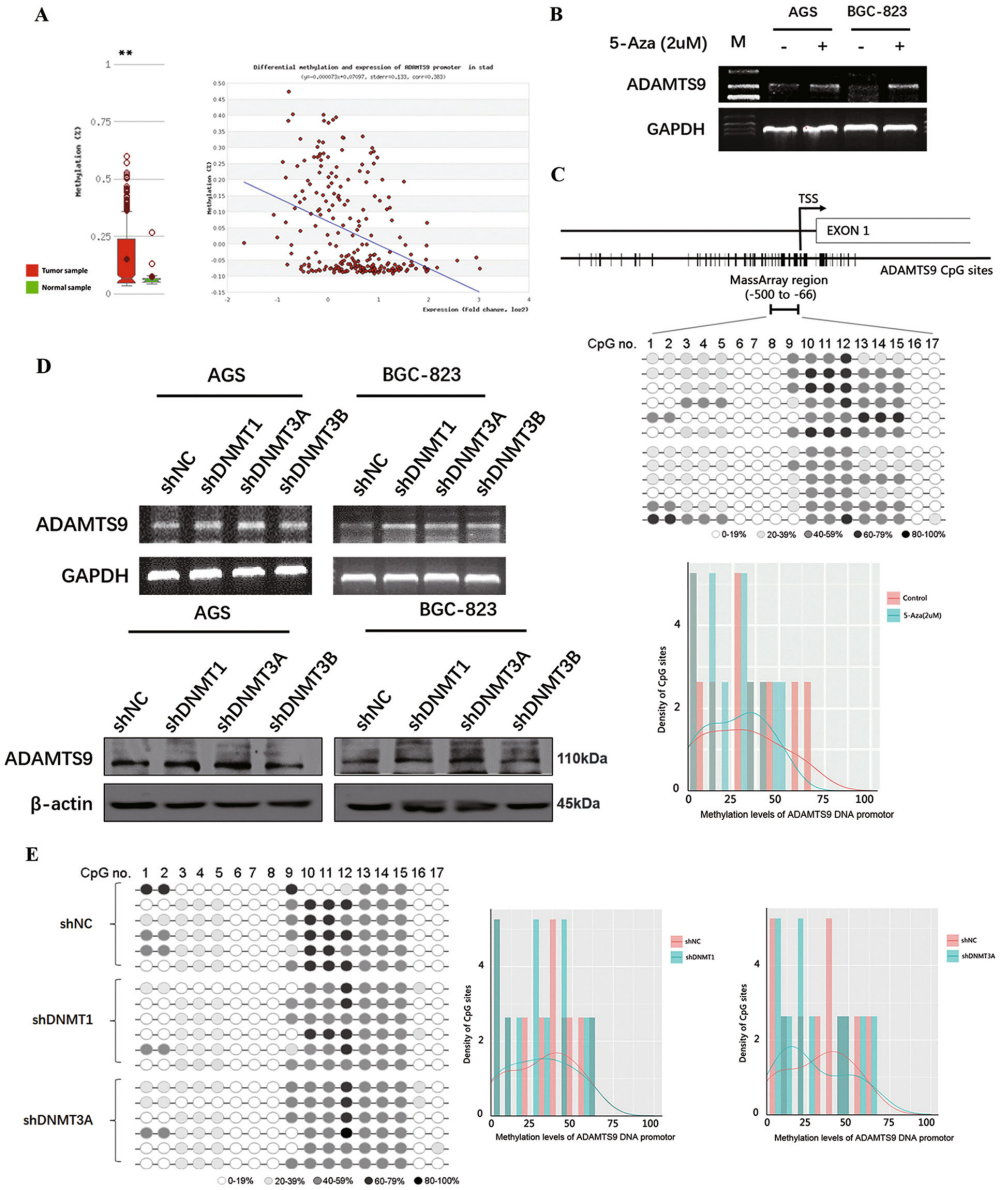
DNA promoter methylation mainly regulated by the DNMT3A may give rise in the low expression of ADAMTS9 in GC cells. **A** ADAMTS9 DNA promotor is hypermethylated in the gastric cancer, according to the MethHC database. **B** The ADAMTS9 expression in AGS and BGC-823 cells after pharmacological reversal of DNA methylation by 5-Aza was examined by reverse transcriptase PCR. **C** The MassARRAY analysis spans the promoter region from −500 to −66, including 17 CpG islands. The methylation levels of ADAMTS9 DNA promotor after 5-Aza incubation were showed. **D** The expression of ADAMTS9 in AGS and BGC-823 cells was examined by reverse transcriptase PCR and western blotting method, after transfection with shDNMT1, shDNMT3A, shDNMT3B, or empty plasmid. **E** The methylation levels of ADAMTS9 DNA promotor after transfection were detected by the MassARRAY analysis.

According to MethHC database (http://methhc.mbc.nctu.edu.tw/php), the ADAMTS9 promotor is hypermethylated in GC, thereby leading to silencing of ADAMTS9 expression (Fig. 4A). Next, we tried to verify this result in current cell model via treating AGS and BGC-823 cells with 5-aza-2′-deoxycytidine (5-Aza). As shown in Fig. 4B, ADAMTS9 mRNA level significantly increased owing to 5-Aza (2 µM) treatment. MassARRAY analysis was then used to examine the methylation of the ADAMTS9 promotor, and demethylation was observed in BGC-823 cells treated with 5-Aza (Fig. 4C and Supplementary Fig. S2A). It is well known that DNMT1, DNMT3A, and DNMT3B primarily contribute to DNA methylation and are closely related to hypermethylation in gene promoters. Therefore, we tried to determine which methyltransferase was indeed the one to maintain densely methylated promoter of ADAMTS9. Specific shRNAs targeting DNMT1, DNMT3A, and DNMT3B were engineered and the knockdown efficiency of the three genes were verified using qPCR and immunoblot (Supplementary Fig. S3). We found that DNMT3A, rather than DNMT1 and DNMT3B, played the major role in sustaining hypermethylation of ADAMTS9 promoter, as decreased DNMT3A increased the ADAMTS9 expression (Fig. 4D). Additionally, it is confirmed that DNMT3A is the methyltransferase that significantly increased methylation level of ADAMTS9 promoter based on MassARRAY platform (Fig. 4E and Supplementary Fig. S2B, S2C).

### RNF180 restores ADAMTS9 expression by promoting ubiquitin-mediated DNMT3A degradation

**Fig. 5:**
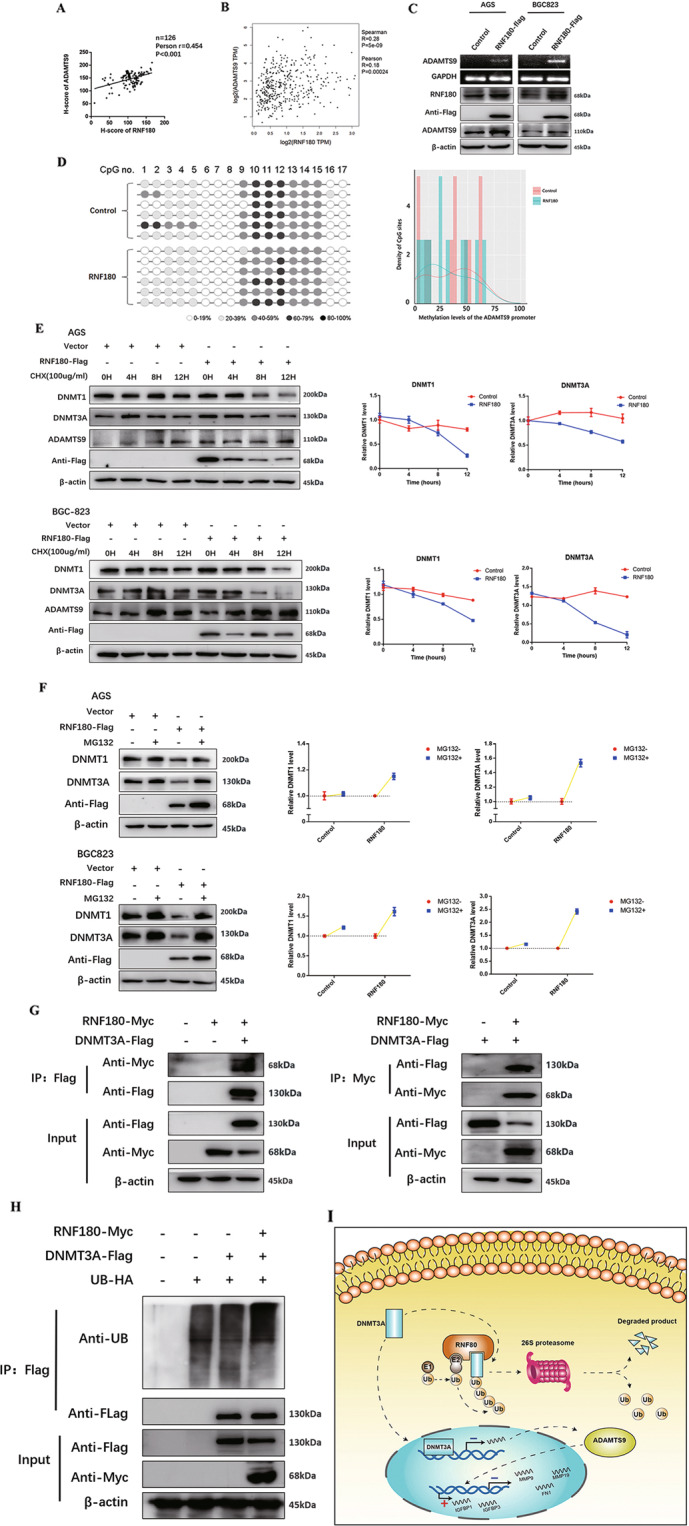
RNF180 restored ADAMTS9 expression in GC cells by promoting DNMT3A protein ubiquitination and degradation. **A** The expression of RNF180 has a significant positive correlation with the expression of ADAMTS9, according to IHC of TMAs; **B** and the online DEPIA database. **C** The expression of ADAMTS9 was examined by PCR and western blotting method, after transfection with RNF180-overexpression plasmid. **D** The methylation levels of ADAMTS9 DNA promotor after transfection were examined by the MassARRAY analysis. **E** AGS and BGC-823 cells were transfected by RNF180-Flag plasmid or control vector. After 48 h, cells were treated with 100 mg/ml CHX at the indicated time point. The DNMT1, DNMT3A, ADAMTS9, and RNF180-Flag proteins were measured through western blotting method. **F** AGS and BGC-823 cells were transfected by RNF180-Flag plasmid or control vector. After 48 h, cells were incubated with 10 uM MG132 for 24 h. The DNMT1, DNMT3A, and RNF180-Flag proteins were measured through western blotting method. **G** RNF180-Myc plasmid or control vector was co-transfected transiently with the DNMT3A-Flag plasmid or control vector into HEK293T cells. After transfection 36 h, HEK293T cells were treated with 10 uM MG132 for 12 h. Then, co-immunoprecipitation assay was performed to pull down DNMT3A-Flag and RNF180-Myc proteins and the immunoprecipitated proteins were measured through western blotting method. **H** UB-HA and DNMT3A-Flag plasmids were co-transfected with RNF180-Myc plasmid into HEK293T cells. After 36 h, HEK293T cells were treated with 10 uM MG132 for 12 h. DNMT3A-Flag protein was immunoprecipitated and measured by western blotting method. The poly-ubiquitination level of DNMT3A was detected by anti-HA antibody. **I** Schematic diagram: RNF180 ubiquitinated DNMT3A by proteasome and restored ADAMTS9 expression through suppressing promotor methylation. ADAMTS9 inhibited MMP9, MMP19, and FN1 transcription and upregulated IGFBP1, and IGFBP3, which impaired the viability and migration behavior of GC.

Our previous studies demonstrated that RNF180, as a tumor suppressor in GC, inhibited proliferation and motility in GC cells^19^, and we wondered whether RNF180 was involved in DNMT3A-ADAMTS9 axis. We first explored the possible correlation between RNF180 and ADAMTS9 in the TMAs described before, finding that significantly positive correlation between RNF180 and ADAMTS9 expression (*N* = 126, Pearson *r* = 0.454, *P* < 0.001; Fig. 5A and Supplementary Fig. S4). In addition, analysis based on data deposited in GEPIA database (http://gepia.cancer-pku.cn/) showed that RNF180 mRNA expression may be positively associated with ADAMTS9 (Spearman *r* = 0.28, *P* = 5e-09; Pearson *r* = 0.18, *P* = 0.00024; Fig. 5B). Thus, we proposed that RNF180 probably restored ADAMTS9 expression by decreasing ADAMTS9 promotor methylation. Results of PCR and immunoblot indicated that RNF180 upregulated ADAMTS9 expression (Fig. 5C). We then performed methylation analysis of ADAMTS9 promoter in BGC-823 cells with RNF180 overexpression, showing that its methylation was markedly reduced (Fig. 5D and Supplementary Fig. S2D). These results together corroborated that RNF180 could increase ADAMTS9 expression by decreasing methylation in ADAMTS9 promotor.

As we had identified that DNMT3A was the major methyltransferase in ADAMTS9 promotor hypermethylation, we next set out to investigate the possible regulation between RNF180 and DNMT3A. Cycloheximide (CHX) was added in AGS and BGC-823 cells and we examined whether RNF180 could affect DNMT1/3A protein stability. We observed that RNF180 promoted degradation of both DNMT1 and DNMT3A protein (Fig. 5E). MG132 (10 µM) was subsequently added in the two cells, eliciting accumulation of both DNMT1 and DNMT3A even when RNF180 was present (Fig. 5F). Moreover, we performed co-immunoprecipitation assays, showing that DNMT3A and RNF180 reciprocally bound to one another while no direct interaction was observed between DNMT1 and RNF180 (Fig. 5G and Supplementary Fig. S5). Finally, ubiquitination assays were performed to validate the level of ubiquitination of DNMT3A. DNMT3A ubiquitination was greatly enhanced as the result of RNF180 overexpression (Fig. 5H). Hence, we confirmed that DNMT3A was a direct substrate of RNF180 and then subject to proteasomal degradation.

## Discussion

In the current study, we provided experimental and clinical evidence to support the suppressive effect of ADAMTS9 on GC. Our study demonstrated that ADAMTS9 inhibited the viability, migration, and invasion of GC cells. Clinical data suggested that the low expression level of ADAMTS9 was closely associated with advanced pN stage and poor survival outcomes and was identified as an independent risk factor. Our findings suggested that ADAMTS9 might be a potential biomarker for predicting the prognosis of GC patients and help improve our understanding of the mechanism of GC development.

Hypermethylation of CpG islands in the promotor region of the ADAMTS9 gene, which was detected by MassARRAY analysis, was closely associated with the absence or downregulation of ADAMTS9 expression. ADAMTS9 expression was largely regulated by methylation of its promotor. These findings were consistent with those previously reported^15^. The silencing or promoting of some tumor-related genes through hypermethylation or hypomethylation plays crucial roles in the occurrence and development of various cancer^20^. Therefore, potential molecular mechanism of promotor methylation of ADAMTS9 should be investigated. In our current study, ADAMTS9 expression was primarily inhibited by DNMT3A through enhanced methylation of the ADAMTS9 gene promotor. DNMT1, DNMT3A, and DNMT3B are three catalytically active DNMTs in mammals that participate in a diverse range of biological processes^21^. For instance, DNMT3A and DNMT3B are responsible for the maintenance of DNA methylation through de novo methylation activity in early embryonic stages and during cell differentiation while DNMT1 is mostly responsible for the maintenance of DNA methylation during replication^22^. Dysregulation of the expression of DNMTs can elicit hypomethylation or hypermethylation of several tumor-related genes through epigenetic changes^23,24^. Through using of the online GEPIA database and IHC staining of 135 GC tissues from our center, we found a significant positive correlation between the expression of RNF180 and ADAMTS9. According to our previous studies, RNF180 is a suppressor gene that inhibits LN metastasis in GC and could act as an independent prognostic indicator of gastric cancer^19,25^. RNF180 is an E3 ubiquitin ligase that belongs to the ubiquitin-proteasome system and plays an important role in post-translational modification, which actually influence several processes of oncogenesis and tumor progression^26^. However, the substrate of RNF180 remains unclear. In the current study, we determined RNF180 could reduce DNMT3A stability through the ubiquitin-proteasome process and identified DNMT3A as the direct substrate of RNF180. As a tumor suppressor, RNF180 could restore the activity of some tumor-related genes, including ADAMTS9, and played a crucial role in impairing lymphatic involvement of GC cells. In summary, ADAMTS9 expression was restored by RNF180-mediated ubiquitination and degradation of DNMT3A.

We further investigated the target genes of ADAMTS9 on GC cell lines and its downstream molecular pathway. ADAMTS9 might attenuate the viability and motile capacity of GC cells, owing to upregulate IGFBP1/3 and downregulate MMP9/19 and FN1. IGFBP1 and IGFBP3 were recognized as tumor suppressor factors in GC. High IGFBP1 was associated with lower GC metastasis and inhibited the tumor cell migration and invasion owing to decreasing MMP9 expression in GC cells^27,28^. IGFBP3 was known to exert insulin-like growth factor (IGF)-independent effects to inhibit cell proliferation and migration, and enhance apoptosis in many malignant tumor cells. IGFBP3 was associated with prognosis of patients with GC, and inhibited proliferation, migration as well as invasion of GC cells by suppressing the NF-κB activity, and inactivating metalloproteinase 14 (MMP14) and urokinase-type plasminogen activator (uPA)^29,30^. Moreover, MMPs, as significant proteases, could cleave ECM and regulate ECM remodeling, which played a crucial role in many biological processes, such as tumourigenesis and wound repair^31,32^. MMPs are now regarded as an important cancer biomarker for tumor invasion and migration. Accumulated evidences have shown upregulated MMP9/19 enhanced the motile capacity of GC cells^33,34^. In addition, FN1 was identified as an important regulatory factor to promote formation and development of diverse tumor cells, such as colorectal cancer and gastric cancer^35,36^. FN1 facilitated proliferation, migration, and invasion of GC cells through activating MMP2/MMP9 pathway^37^. In this study, we offered some explanation for anti-proliferation and anti-invasion/migration effect of ADAMTS9 by upregulating IGFBP1/3 and downregulating MMP9/19 and FN1.

In conclusion, we uncovered RNF180/DNMT3A/ADAMTS9 axis in GC cells and showed how the signaling pathway impacted GC cells. In addition, low ADAMTS9 expression was closely associated with advanced pN stage and poor survival outcome and might serve as a biomarker to predict metastatic risk in patients with GC.

## Supplementary information

Supplementary tables

Supplementary dataset

Supplementary Figure and table legends

Supplementary Figure S1

Supplementary Figure S2

Supplementary Figure S3

Supplementary Figure S4

Supplementary Figure S5

## Data Availability

The RNA-sequencing data can be obtained from the NCBI Sequence Read Archive (BioProject ID PRJNA644154).
