# Supplementary material for: DNMT3A-mediated silence in ADAMTS9 expression is restored by RNF180 to inhibit viability and motility in gastric cancer cells
Source: Cell Death Dis. 2021 Apr 30;12(5):428. doi: 10.1038/s41419-021-03628-5 (PMC8087691; doi:10.1038/s41419-021-03628-5)
Supplement: Supplementary file 1 — Supplementary tables [file 41419_2021_3628_MOESM1_ESM.doc]

**Supplementary Table S1.** Clinicopathologic Features of Gastric Cancer Patients with ADAMTS9 mRNA Expression

Note:

| **Characteristics** | | **ADAMTS9  high expression** | **ADAMTS9 low expression** | **χ^2^ value** | **P value** |
| --- | --- | --- | --- | --- | --- |
| Gender | |  |  |  |  |
|  | Male | 5 | 4 | 1.165 | 0.568^a^ |
|  | Female | 2 | 5 |  |  |
| Age | |  |  |  |  |
|  | <60 | 2 | 3 | 0.042 | 1.000^a^ |
|  | ≥60 | 5 | 6 |  |  |
| Tumor size | |  |  |  |  |
|  | <5cm | 5 | 6 | 0.042 | 1.000^a^ |
|  | ≥5cm | 2 | 3 |  |  |
| pT stage | |  |  |  |  |
|  | pT1-pT3 | 4 | 6 | 0.152 | 1.000^a^ |
|  | pT4 | 3 | 3 |  |  |
| pN stage | |  |  |  |  |
|  | pN0-pN2 | 6 | 2 | 6.349 | 0.044^a*^ |
|  | pN3 | 1 | 7 |  |  |
| pTNM stage | |  |  |  |  |
|  | I-II | 3 | 1 | 2.116 | 0.383^a^ |
|  | III | 4 | 8 |  |  |
| lauren type | |  |  |  |  |
|  | Intestinal | 4 | 0 | 6.857 | 0.019^b*^ |
|  | Diffuse | 3 | 9 |  |  |

^a^, continuity correction test;

^b^, fisher exact test;

^*^, P<0.05.

**Supplementary Table S2.** Clinicopathologic Features of ADAMTS9 Expression in Gastric Cancer Tissues detected by immunohistochemistry

| **Characteristics** | | **ADAMTS9  high expression** | **ADAMTS9  low expression** | **χ^2^ value** | **P value** |
| --- | --- | --- | --- | --- | --- |
| **Gender** | |  |  |  |  |
|  | Male | 45 | 45 | 0.018 | 0.894 |
|  | Female | 19 | 20 |  |  |
| **Age** | |  |  |  |  |
|  | <60 | 33 | 37 | 0.373 | 0.541 |
|  | ≥60 | 31 | 28 |  |  |
| **Tumor location** | |  |  |  |  |
|  | Upper third | 13 | 10 | 1.83 | 0.608 |
|  | Middle third | 8 | 5 |  |  |
|  | Lower third | 29 | 36 |  |  |
|  | More than 2/3 stomach | 14 | 14 |  |  |
| **Tumor size** | |  |  |  |  |
|  | <5cm | 25 | 27 | 0.082 | 0.774 |
|  | ≥5cm | 39 | 38 |  |  |
| **pT stage** | |  |  |  |  |
|  | pT2 | 5 | 4 | 0.326 | 0.85 |
|  | pT3 | 4 | 3 |  |  |
|  | pT4 | 55 | 58 |  |  |
| **pN stge** | |  |  |  |  |
|  | pN0 | 18 | 10 | 10.043 | 0.018^*^ |
|  | pN1 | 9 | 4 |  |  |
|  | pN2 | 15 | 11 |  |  |
|  | pN3 | 22 | 40 |  |  |
| **pTNM stage** | |  |  |  |  |
|  | I | 3 | 1 | 2.48 | 0.285^b^ |
|  | II | 18 | 13 |  |  |
|  | III | 43 | 51 |  |  |
| **Lauren type** | |  |  |  |  |
|  | Intestinal | 13 | 12 | 3.066 | 0.216 |
|  | Diffuse | 46 | 52 |  |  |
|  | Mixed | 5 | 1 |  |  |
| **Borrman type^1^** | |  |  |  |  |
|  | Ⅰ | 0 | 1 | 3.271 | 0.352 |
|  | Ⅱ | 9 | 14 |  |  |
|  | Ⅲ | 33 | 25 |  |  |
|  | Ⅳ | 5 | 6 |  |  |

Note：^1^, some data has lost; ^a^, continuity correction test; ^b^, fisher exact test;

^*^, P<0.05;

**Supplementary Table S3.** Main antibodies adopted in study

| Name | Company | Cat. No. | Concentration |
| --- | --- | --- | --- |
| anti-ADAMTS9 | Abcam | ab32565 | 1:1000(WB) |
| anti-ADAMTS9 | Abcam | ab224132 | 1:50(IHC) |
| anti-DNMT1 | Cst | D63A6 | 1:1000(WB) |
| anti-DNMT3A | Cst | D23G1 | 1:1000(WB) |
| anti-DNMT3B | Cst | D7O7O | 1:1000(WB) |
| anti-RNF180 | GeneTex | GTX119301 | 1:1000(WB),1:100(IHC) |
| anti-Flag-Tag | Cst | D6W5B | 1:1000(WB) |
| anti-Myc-Tag | Cst | #2272 | 1:1000(WB) |
| anti-HA-Tag | Cst | C29F4 | 1:1000(WB) |
| anti-β-actin | Cst | 1.30E+06 | 1:1000(WB) |

**Supplementary Table S4.** Real-time PCR primers in study

| Gene | type | Sequence (5’ to 3’) |
| --- | --- | --- |
| IGFBP1 | Forwrd primer | TTGGGACGCCATCAGTACCTA |
| IGFBP1 | Reverse primer | TTGGCTAAACTCTCTACGACTCT |
| IGFBP3 | Forwrd primer | CCGCCAGCTCCAGGAAAT |
| IGFBP3 | Reverse primer | GGGGTGGAACTTGGGATCAG |
| MMP9 | Forwrd primer | TGTACCGCTATGGTTACACTCG |
| MMP9 | Reverse primer | GGCAGGGACAGTTGCTTCT |
| MMP19 | Forwrd primer | GCTTCCTACTCCCCATGACAG |
| MMP19 | Reverse primer | CCCATATTGTGACAGGTAGTCCA |
| FN1 | Forwrd primer | CGGTGGCTGTCAGTCAAAG |
| FN1 | Reverse primer | AAACCTCGGCTTCCTCCATAA |
| DNMT1 | Forwrd primer | TACCTGGACGACCCTGACCTC |
| DNMT1 | Reverse primer | CGTTGGCATCAAAGATGGACA |
| DNMT3A | Forwrd primer | TATTGATGAGCGCACAAGAGAGC |
| DNMT3A | Reverse primer | GGGTGTTCCAGGGTAACATTGAG |
| DNMT3B | Forwrd primer | GGCAAGTTCTCCGAGGTCTCTG |
| DNMT3B | Reverse primer | TGGTACATGGCTTTTCGATAGGA |
| HPRT1 | Forwrd primer | TGACACTGGCAAAACAATGCA |
| HPRT1 | Reverse primer | GGTCCTTTTCACCAGCAAGCT |
