# Supplementary material for: DNMT3A-mediated silence in ADAMTS9 expression is restored by RNF180 to inhibit viability and motility in gastric cancer cells
Source: Cell Death Dis. 2021 Apr 30;12(5):428. doi: 10.1038/s41419-021-03628-5 (PMC8087691; doi:10.1038/s41419-021-03628-5)
Supplement: Supplementary file 2 — Supplementary dataset [file 41419_2021_3628_MOESM2_ESM.doc]

**Supplementary dataset:**

**1.Cell lines:**

Five GC cell lines, namely, KATO III, SNU-1, SNU-16, NCI-N87, and AGS, were obtained from the American Type Culture Collection (ATCC, Manassas, VA, USA). Five GC cell lines, namely, MKN-45, HGC-27, BGC-823, MGC-803, and SGC-7901, and normal human gastric epithelial cell line (GES-1) were obtained from Cancer Research Institute of Beijing, Beijing University, China. All GC and GES-1 cell lines, excluding KATO III and AGS cell lines were cultured in RPMI 1640 medium (Gibco, Carlsbad, CA, USA) supplemented with 10% fetal bovine serum. The KATO III was cultured in Iscove’s Modified Dulbecco's Medium (Gibco, Carlsbad, CA, USA), with 20% fetal bovine serum. AGS was cultured in F12 Nutrient Mixture medium (Gibco, Carlsbad, CA, USA) supplemented with 10% fetal bovine serum. HEK293T cell line was obtained from ATCC and cultured in Dulbecco's Modified Eagle’s medium supplemented with 10% fetal bovine serum. All cell lines were maintained at 37 °C in a humidified atmosphere at 5% CO_2_ and 95% air.

**2.Plasmid and transfection**

The pCDNA3.1-ADAMTS9 plasmid was a gift from Professor Lung, Maria Li (the Department of Clinical Oncology, Li Ka Shing Faculty of Medicine, Chinese University of Hong Kong, Hong Kong, China). The pCDNA3.1-ADAMTS9 plasmid encodes the full-length human ADAMTS9 complementary DNA (NM_182920), which was sequence-verified. The following shRNA sequences and plasmids in this study were obtained from Shanghai GeneChem Co. (Shanghai, China): shDNMT1 (5′-gcCCAATGAGACTGACATCAA-3′), shDNMT3A (5′-ccGGCTCTTCTTTGA GTTCTA-3′), shDNMT3B (5′-AGATGACGGATGCCTAGAG-3′), pCMV-RNF180, pCMV-RNF180-Myc, pCMV-RNF180-Flag, and pCMV-DNMT3A-Flag plasmids. The plasmid pCMV-HA-UB was obtained from the Ke Lei Biological Technology Co. (Shanghai, China). Plasmids were transfected with Lipofectamine TM 3000 transfection reagent (Invitrogen, New York, USA) and Opti-MEM (Invitrogen, New York, USA). The stable transfected cells were selected from G418 or puromycin treatment.

**3.RNA extraction and PCR analyses**

Total RNA was extracted from cell lines by using Trizol reagent (Invitrogen, CA, USA). Complementary DNA was synthesized from total RNA by using PrimeScript™ RT Master Mix (TaKaRa, Shiga, Japan) according to the manufacturer’s instructions. For semiquantitative reverse-transcription–PCR, 2* Taq PCR MasterMix (TIANGEN Biotech, Beijing, China) was used for amplifying ADAMTS9 and glyceraldehyde-3-phosphate dehydrogenase (GAPDH) gene according to the manufacturer's protocol. The PCR for ADAMTS9 gene was performed under the following conditions: 3 min at 94 °C, 30 cycles of 30 s at 94 °C, 30 s at 53 °C, and 1 min at 72 °C; and the final 5 min at 72 °C. The PCR for GAPDH gene was performed under the same conditions but at 50 °C annealing temperature. Primers designed and utilized for ADAMTS9 were 5′-TTAATCTCACCGCCAATGCC-3′ and 5′-GCGCTGCGCCTATAAATGAT-3′. The primers for GAPDH were 5′-GAGTCAACGGATTTGGTCGT-3′ and 5′-ATCCACAGTCTTCTGGGTGG-3′. SYBR Green PCR Master Mix (TaKaRa, Shiga, Japan) was used for Real-time PCR. Primers for real-time PCR in this study are shown in the **Supplementary Table S4,** and the HPRT1 gene served as the endogenous control.

**4.Western blot analysis**

Total protein was extracted by the RIPA buffer (#9806, cst) supplemented with phenylmethanesulfonyl fluoride (PMSF, #8553, cst). Approximately 20 ug of protein was separated by the SDS-PAGE and transferred onto nitrocellulose membrane. The primary antibodies in this study are shown in **Supplementary Table S3**. AI600 Imager system (GE Healthcare, NJ, US) was used to screen the target protein and to determine gray values.

**5.CCK8 Assay**

CCK8 assay was applied to evaluate the effects of ADAMTS9 in the proliferation and viability of GC cells. AGS, BGC-823, and SGC-7901 cells stably transfected with pCDNA3.1-ADAMTS9 and pCDNA3.1-empty vector were seeded (2000 cells per well) in the 96-well plates and measured once a day. Approximately 10 ul of CCK8 solution was added to every single well in the plate and was incubated for 2 h at 37 °C. After incubation, the absorbance was measured at 450 nm in a spectrophotometer. The assay was replicated three times.

**6.Colony formation assay**

Stable transfected cells (300 cells per well) were seeded in the 6-well plate and incubated for 1 week. Colonies with > 50 cells per colony were counted after staining with crystal violet solution. The assay was replicated three times.

**7.*In vivo* tumorigenicity**

The BGC-823 cells (1×10^6^ cells in 0.1 ml phosphate-buffered saline) stably transfected with pCNDA3.1-ADAMTS9 vector or pCDNA3.1 empty vector were separately injected subcutaneously into the dorsal flanks of 6 5-week-old male Bal b/c nude mice. Tumor volume (mm^3^) was estimated by measuring the longest and the shortest diameters of the tumor and calculated as follows: volume = (shortest diameter)^2^
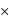
(longest diameter)
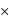
0.5. The investigator was blinded to the tumor sites during estimating the tumor volume and weight. Before injecting tumor cells into mice, we administered tribromoethanol (350mg/kg) to the mice by intraperitoneal injection. The permission for animals was given by the Institutional Research Ethics Committee of Tianjin Medical University Cancer Institute and Hospital.

**8.Wound healing assay**

Stable transfected AGS, BGC-823 and SGC-7901 cells were seeded in the 6-well plates and grown to confluency. A scratch was made with a 200 µL pipette tip per dish, and the scratch width was measured as the baseline. The width of the scratches was measured using the Q capture Pro software every 12 h. Percentage wound healing was calculated, and the results presented are mean ± standard deviation. The assay was replicated three times.

**9.Transwell assay**

The 24-well Transwell chambers (8.0 μm pore size; Corning Costar, Cambridge, MA) were used to perform cell invasion assay with Matrigel (100 μl of serum free medium with 2.6 μl of Matrigel, BD Biosciences, San Jose, CA) and migration assay without Matrigel. Approximately 100 μl of serum-free medium with 4×10^5^/mL stable transfected cells was seeded into the upper chamber, whereas the medium with 20% FBS as a chemoattractant was added into the lower chamber. After 24 h of incubation, non-invaded cells were removed using cotton swabs. The cells on the underside of chambers were fixed in methanol for 30 min. The invaded cells were then stained with crystal violet solution for 2 min. The assay was replicated three times.

**10. Immunohistochemistry (IHC)**

IHC was performed on the TMAs of resected specimens. The 1.5 mm-diameter tissue cores from randomly selected GC tissues and matched adjacent non-tumor tissues were used for TMA preparation. The antibodies are as shown in Supplementary Table S3. The staining intensities (0, negative; 1+, weak; 2+, moderate; and 3+, strong) were recorded. The percentage of immunostaining and the cytoplasmic expression were assessed by H-score system. The formula for the H-score is: histoscore = Σ (I × Pi), where I = intensity of staining and Pi = percentage of stained tumor cells, producing a cytoplasmic score ranging from 0 to 300.

**11. Cycloheximide (CHX) pulse-chase assay and MG132 assay**

To detect the half-life of DNMT3A, we treated the stable transfected AGS and BGC-823 cells with CHX (100 mg/mL) for the indicated times. MG132 assay was performed to determine the degradation via proteasome. AGS and BGC-823 cells were incubated with proteasome inhibitor MG132 (10 µM) for 12 h. Western blot analysis was then performed to detect DNMT3A expressions.

**12. Co-immunoprecipitation assay**

HEK293T cells were seeded into 6-well plates and transiently transfected with empty vector, pCMV-RNF180-myc plasmid, or pCMV-DNMT3A-flag plasmid. After transfection for 36 h, cells were treated with 10 mM MG132 for 12 h. Total proteins were then extracted using Nonidet P-40 lysis buffer supplemented with PSMF (#8553, cst). Immunoprecipitation Kit-DYKDDDDK (Flag®) Tag Immunomagnetic Beads (TB101274, Sino Biological, North Wales, PA, USA) and Immunoprecipitation Kit -MYC Tag Immunomagnetic Beads (TB100029, Sino Biological, North Wales, PA, USA) were adopted for collecting the immunoprecipitated proteins. The immunoprecipitated proteins were measured by the Western blot analysis.

**13. Ubiquitination assay**

To evaluate the ubiquitination of DNMT3A, we transiently transfected empty vector, pCMV-DNMT3A-flag plasmid, pCMV-RNF180-myc plasmid or pCMV-HA-UB plasmid the HEK293T cells. After transfection for 36 h, HEK293T cells were incubated with MG132 (10 µM) for 12 h. The correlated proteins were extracted and analyzed by the co-immunoprecipitation assay.

**14. Statistical analysis**

The statistical software package SPSS 24.0 was used for all analyses. Categorical data and continuous variables in the clinicopathological characteristics were analyzed by the χ2 test and Student’s test, respectively. The mean and standard deviation were used to evaluate data. The variables at P<0.05 were included in multivariate logistic regression analysis. Overall survival (OS) was determined using Kaplan–Meier method, and log-rank test was performed to determine significance. Potential factors that have significant effect on OS in univariate analyses (P<0.05) were included in multivariate analysis. The multivariate analysis of OS was performed by the Cox proportional hazard model with forward step procedures. Hazard ratios (HR) and 95% CI were generated. Significance was defined as two-side P<0.05.
