# Supplementary material for: DNMT3A-mediated silence in ADAMTS9 expression is restored by RNF180 to inhibit viability and motility in gastric cancer cells
Source: Cell Death Dis. 2021 Apr 30;12(5):428. doi: 10.1038/s41419-021-03628-5 (PMC8087691; doi:10.1038/s41419-021-03628-5)
Supplement: Supplementary file 3 — Supplementary Figure and table legends [file 41419_2021_3628_MOESM3_ESM.docx]

**Supplementary figure legends：**

**Supplementary Figure S1**. ADAMTS9 attenuated malignant phenotypes in SGC-7901 cells. (A) mRNA and protein expression levels of ADAMTS9 SGC-7901 cells transfected with ADAMTS9 overexpression plasmid and empty plasmid were detected by reverse transcriptase PCR and western blotting method . (B) ADAMTS9 inhibits the cell growth in SGC-7901 cells. (C) ADAMTS9 suppresses the colony formation in SGC-7901 cells. (D) ADAMTS9 impairs motile capacity in SGC-7901 cell, detected by wound healing assay (magnification 40×); (E) and by transwell assay (magnification 100×).

**Supplementary Figure S2**. Methylation levels of CpG islands in ADAMTS9 DNA promotor were examined according to MassARAAY analysis. (A) After incubation with 5-Aza, the significant decreased methylation levels were detected in the CpG island 10, 11 and 12. (B) The CpG island 10 and 11 are significantly hypomethylated through suppressing the DNMT1; (C) and DNMT3A. (D) RNF180 downregulates the methylation levels in the CpG island 10 and 11 in ADAMTS9 DNA promotor.

**Supplementary Figure S3**. The mRNA and protein expression of DNMT1, DNMT3A, and DNMT3B in AGS and BGC-823 cells were confirmed by the qPCR and western blotting method.

**Supplementary Figure S4**. Immunohistochemical staining for RNF180 and ADAMTS9 proteins in TMAs showed that the protein expression of RNF180 was positively associated with ADAMTS9 protein expression.

**Supplementary Figure S5**. RNF180-Myc plasmid was co-transfected transiently with the DNMT1-Flag plasmid or control vector into HEK293T cells. After transfection 36h, HEK293T cells were treated with 10 uM MG132 for 12h. Then, Co-immunoprecipitation assay was performed to pull down DNMT1-Flag and RNF180-Myc proteins and the immunoprecipitated proteins were measured through western blotting method.

**Supplementary table legends:**

**Supplementary Table S1.** Clinicopathologic Features of Gastric Cancer Patients with ADAMTS9 mRNA Expression

**Supplementary Table S2.** Clinicopathologic Features of ADAMTS9 Expression in Gastric Cancer Tissues detected by immunohistochemistry

**Supplementary Table S3.** Main antibodies adopted in study

**Supplementary Table S4.** Real-time PCR primers in study
