# Supplementary figures and images for: DNMT3A-mediated silence in ADAMTS9 expression is restored by RNF180 to inhibit viability and motility in gastric cancer cells
Source: Cell Death Dis. 2021 Apr 30;12(5):428. doi: 10.1038/s41419-021-03628-5 (PMC8087691; doi:10.1038/s41419-021-03628-5)

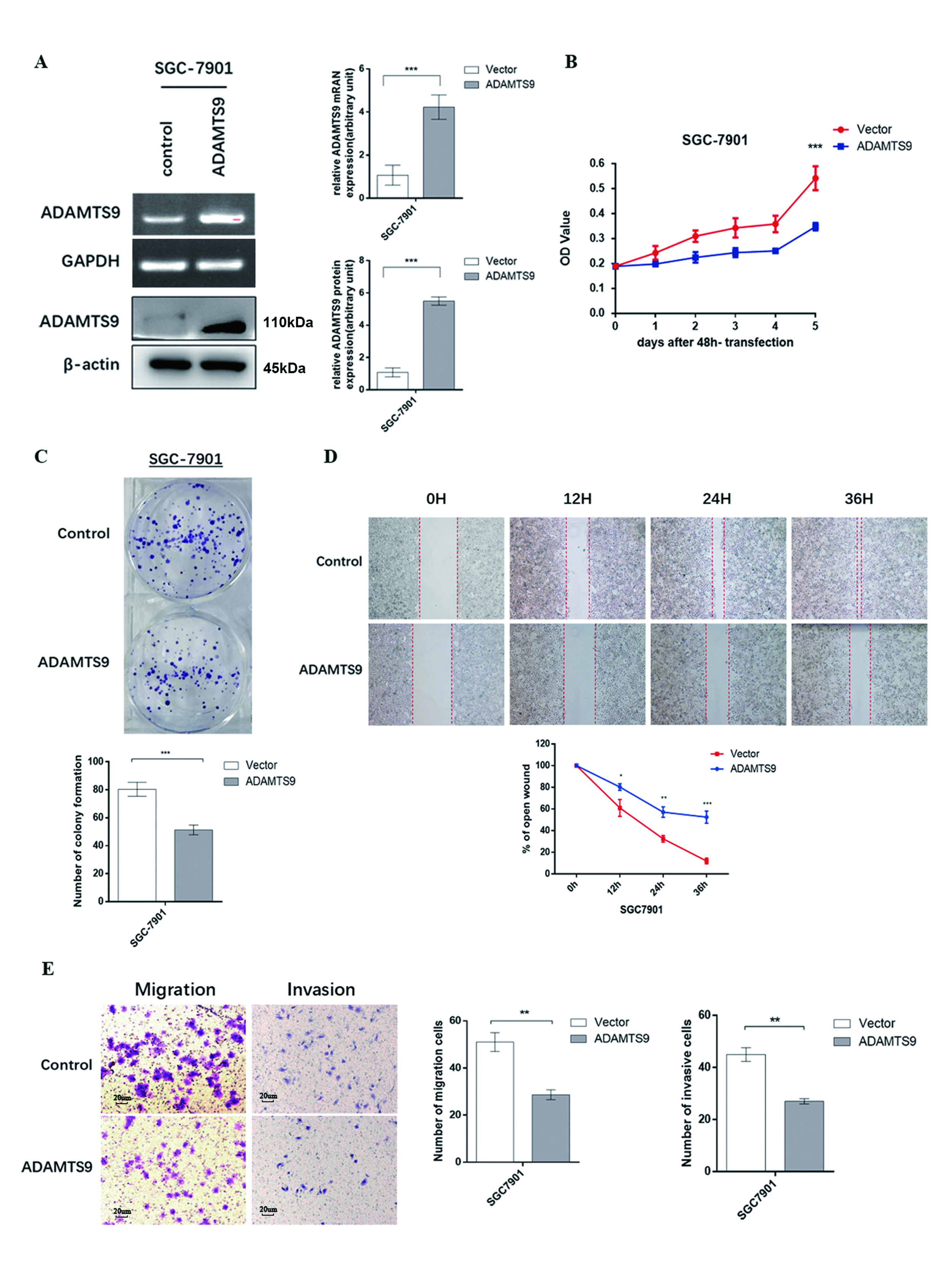

Supplement: Supplementary file 4 — Supplementary Figure S1 [file 41419_2021_3628_MOESM4_ESM.tif]

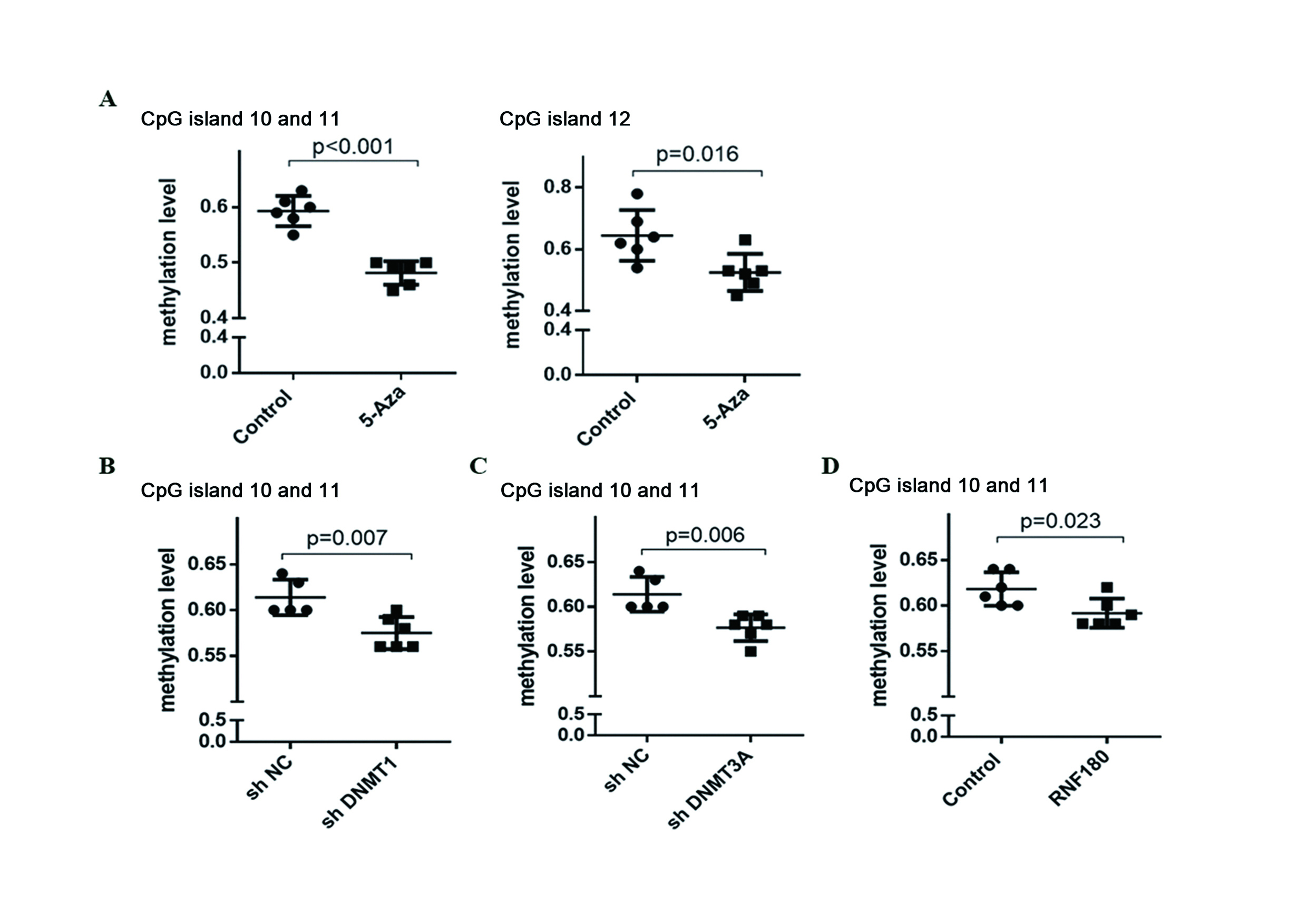

Supplement: Supplementary file 5 — Supplementary Figure S2 [file 41419_2021_3628_MOESM5_ESM.tif]

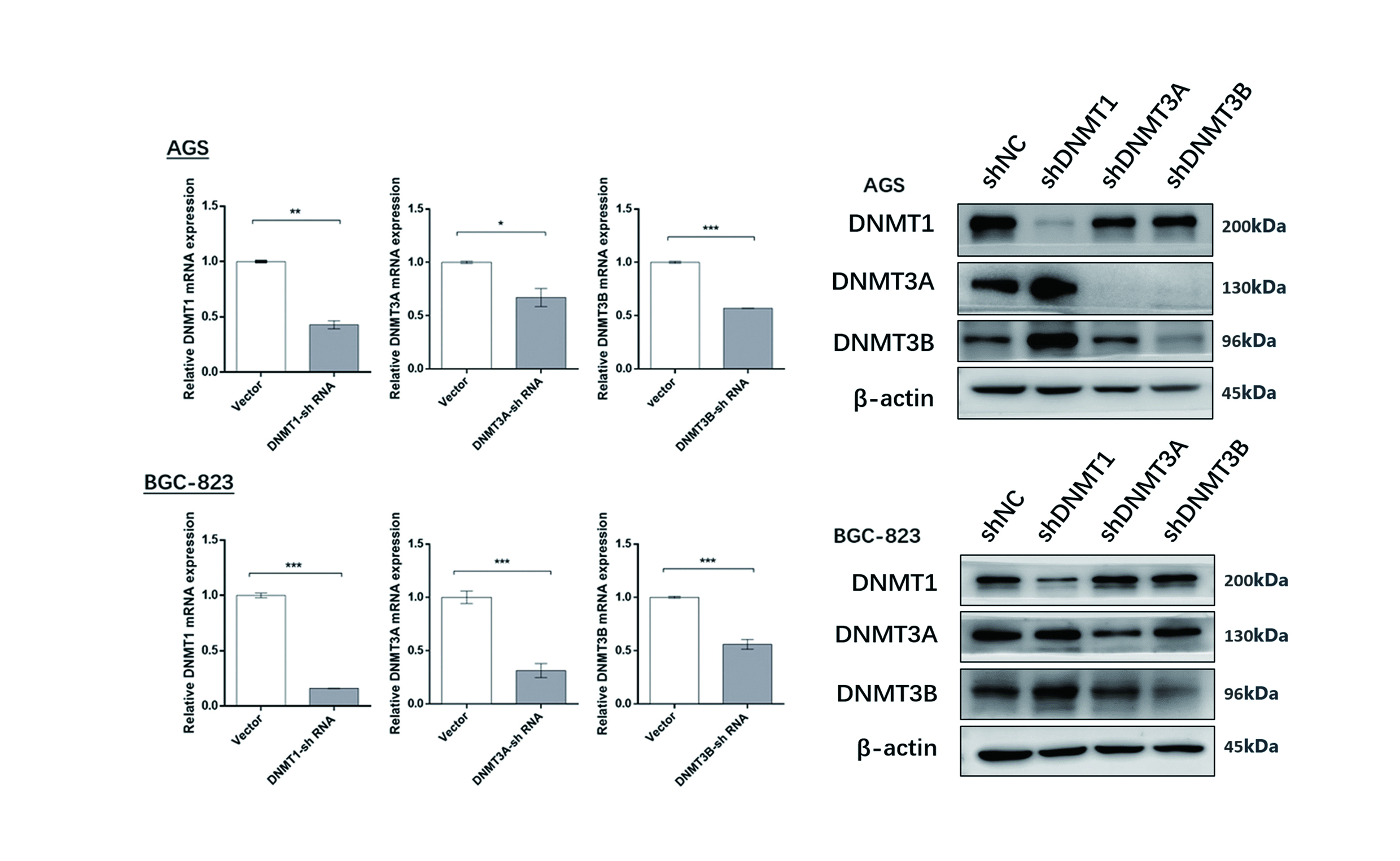

Supplement: Supplementary file 6 — Supplementary Figure S3 [file 41419_2021_3628_MOESM6_ESM.tif]

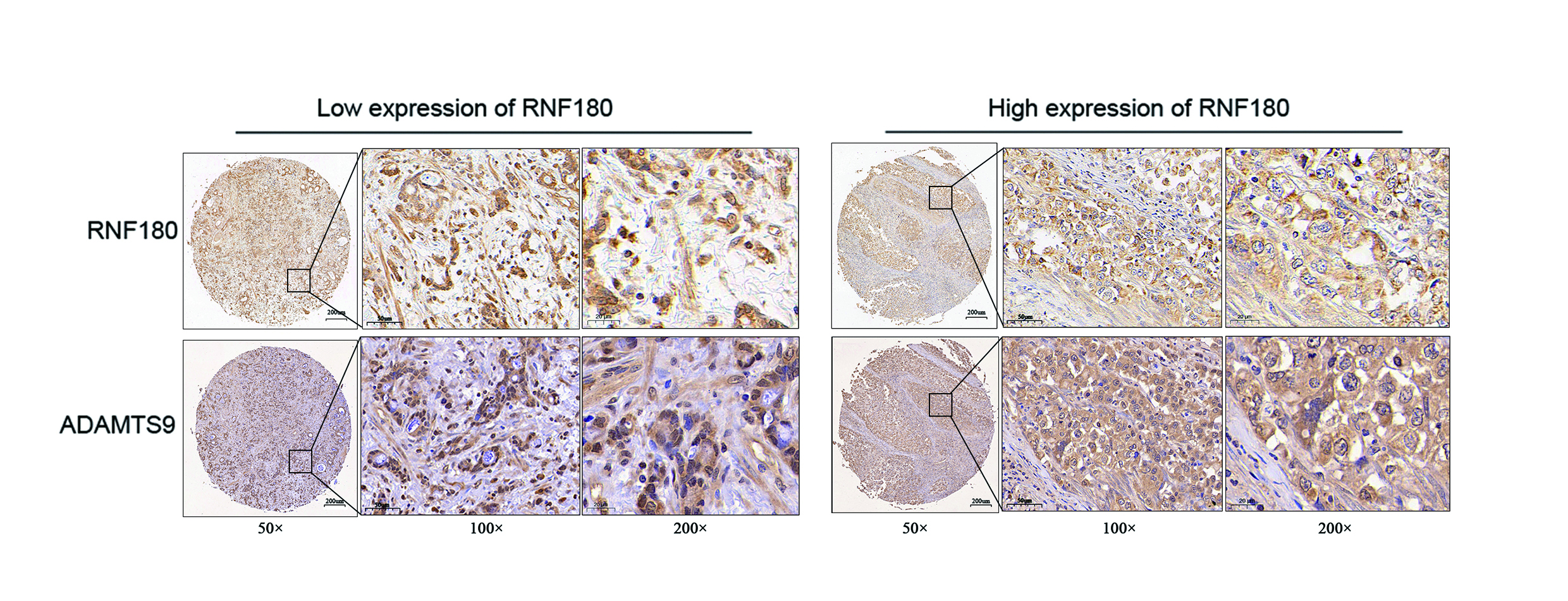

Supplement: Supplementary file 7 — Supplementary Figure S4 [file 41419_2021_3628_MOESM7_ESM.tif]

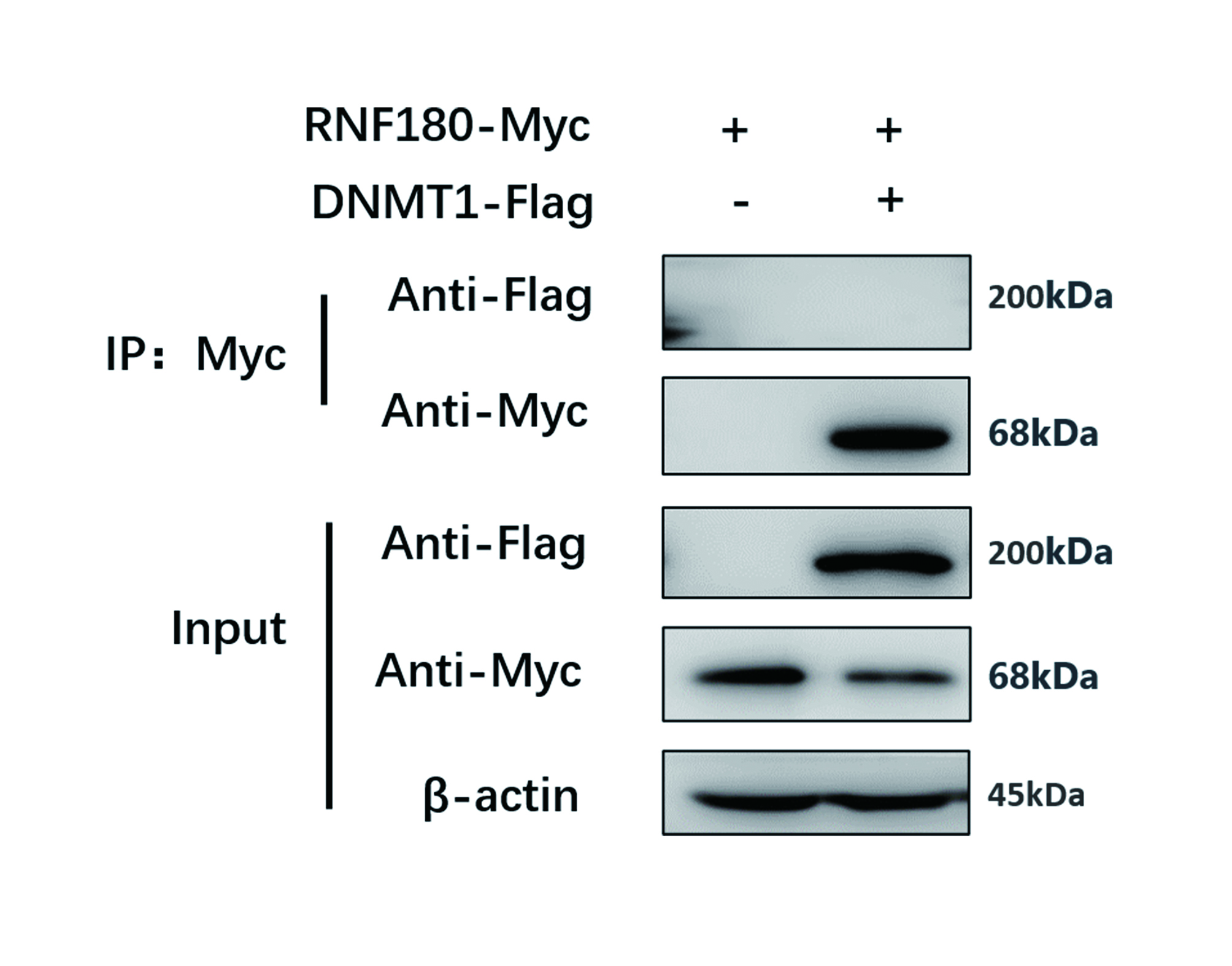

Supplement: Supplementary file 8 — Supplementary Figure S5 [file 41419_2021_3628_MOESM8_ESM.tif]
